# Exploring the prevalence of gout among underrepresented low socioeconomic status type 2 diabetes populations

**DOI:** 10.1186/s13098-025-01586-y

**Published:** 2025-01-21

**Authors:** Jessica Smith, Abey Martin, Jane Mundadan, Michael Roberts, Youssef Roman, Arturo Bravo-Nuevo, Farzaneh Daghigh

**Affiliations:** 1 Philadelphia College of Osteopathic Medicine, Philadelphia, USA; 2 Michigan State University College of Osteopathic Medicine, East Lansing, USA; 3 Idaho State University, Pocatello, USA

**Keywords:** Type 2 Diabetes (T2D), Low socioeconomic status, Hyperuricemia, Gout

## Abstract

**Background:**

Underserved and underrepresented populations often lack access to affordable, quality healthcare, educational resources, and nutritious foods, all of which contribute to increased risk of Type 2 Diabetes and gout. Type 2 Diabetes is a condition characterized by the denaturation of the insulin receptors, due to chronically high blood glucose levels, leading to impaired regulation of blood sugar. Gout is a chronic inflammatory disease affecting joints in the lower limbs, marked by elevated serum urate levels and the accumulation of uric acid crystals in synovial fluid, causing painful flare-ups that significantly impact quality of life.

**Methods:**

This multisite cross-sectional study was conducted in three low-income senior residential communities across the mid-Atlantic United States, including Philadelphia and Harrisburg, Pennsylvania, and Clinton, Maryland. A total of 88 consenting participants were surveyed on their health history and tested for hemoglobin A1c (HbA1c), blood glucose, and uric acid levels using finger-stick blood tests and commercially available devices. Inclusion criteria included individuals of any gender, aged 35–92, residing in these communities. Exclusion criteria were a personal history of cancer, organ transplantation, or current pregnancy. Educational materials were provided after discussing each participant’s results.

**Results:**

There is an identifiable prevalence of gout among this population of low-income senior adults living with Type 2 Diabetes. Among the participants, 30.7% had serum urate levels indicative of hyperuricemia, exceeding the national average of 20.1% as reported by the National Health and Nutrition Examination Survey. Participants with high HbA1c had significantly higher uric acid levels compared to those with lower HbA1c levels, with diabetic levels of HbA1c accounting for approximately 40% of the variance in uric acid levels. Additionally, study participants who smoked cigarettes were more likely to have hyperuricemia than non-smokers.

**Conclusion:**

Preventive educational efforts focused on diet and lifestyle are critical to reducing the incidence of gout and Type 2 Diabetes in low-income elderly populations. Diabetic individuals are at a higher risk of developing hyperuricemia and gout compared to non-diabetics. Community-based educational health programs are necessary to make a measurable impact on these populations, prevent disease progression, and reduce the burden on healthcare systems.

## Background

Diabetes is a chronic disease characterized by reduced glucose uptake from the bloodstream [1]. This could be due to a genetic abnormality in the functioning of β cells in the pancreas that produce insulin, known as Type 1 Diabetes, or the denaturation of the insulin receptor, known as Type 2 Diabetes (T2D) [1]. The development of T2D is frequently associated with obesity, poor diet, and a genetic predisposition. Screening for diabetes involves measuring blood glucose, which varies with food consumption, and Hemoglobin A1c (HbA1c), representing an individual’s circulating glucose level over the past three months [1]. An HbA1c level below 5.7% is within normal limits, while a level above 6.5% requires medical intervention [2].

In recent decades, there has been a marked increase in the clinical investigation of the association between diabetes and gout. Gout is a common form of inflammatory arthritis that primarily presents in the big toe, often termed podagra. It is a result of chronic hyperuricemia which produces an accumulation of monosodium urate crystals in joints and synovial fluid, primarily affecting the joints in the lower limbs [3]. High serum urate concentration is the most direct risk factor for the development of gout. Proteins, including red meats and seafood, are high in purines, which when metabolized by the body, produce uric acid [3]. Alcohol, especially beer, and beverages with a high concentration of sugar, also increase serum urate levels and increase an individual’s risk for gout [4]. Gout can present as either periodic flare-ups, or as chronic gouty arthritis [3], caused by the body’s innate immune response against the monosodium urate crystals. Gout can be an incredibly painful inflammatory condition that affects individuals’ work productivity, quality of life, mental health, and has a negative social stigma [3]. The current treatment recommendations for gout management include long-term urate-reducing therapies, and prevention of gout flares through lifestyle and dietary changes [3].

One major factor that has contributed to the diabetes epidemic is medication non-adherence and patient concerns regarding adverse drug effects. This stems from a lack of understanding due to language barriers, ineffective education, and lack of or reduced access to trustworthy healthcare resources [5]. This coincides with the increasing incidence of gout amongst patients of low-income socioeconomic backgrounds [5]. In a cross-sectional study, gout was found to be more prevalent among black men and women when compared to their white counterparts after adjusting for poverty, diet, body mass index, and chronic kidney disease [6]. This suggests that racial disparities are in fact a major player in the development of gout and may be explained by social determinants of health [6].

Underserved and underrepresented communities in the United States often lack access to quality healthcare and affordable, nutritious food, contributing to the increased prevalence and incidence of T2D and gout in these populations [5]. Risk factors for both diabetes and gout are particularly prominent in racial minorities and low-income communities [6]. Limited access to quality, consistent medical care can cause gout to go undiagnosed and/or untreated [5]. For this reason, investigators in this study partnered with Beacon Communities LLC, a low-income housing community [7]. Our study is centered on three distinct low-income senior housing communities in Philadelphia, PA, Harrisburg, PA, and Clinton, MD. Park Towers Apartments and Branchwood Towers describe themselves as “an affordable community of low-income apartments for seniors 62 + or those who are disabled” [8]. Pheasant Hill Estates describes itself as “specifically designed for qualified seniors 62 years of age or older who have low to moderate incomes, as well as people younger than 62 years of age who are disabled” [9]. The majority of residents within these communities identify as non-Hispanic Black or African American (67%). Most residents have completed a high school education. 100% of residents living within these communities receive public assistance. These locations were selected due to their unique demographics and challenges in healthcare access. Residents in these communities come from diverse backgrounds and are typically known for leading active and independent lives, emphasizing the importance of understanding the health dynamics within these specific populations.

We have formulated a study focused on the intersection between T2D, hyperuricemia, and gout, to investigate the prevalence of gout and diabetes among this population, and provide insight into the correlation between gout and diabetes mellitus. Overall, the objective of this study is to increase the representation of population groups that are historically underrepresented in chronic disease research while having the highest disease burden of both conditions. We hypothesized that there was a higher frequency of T2D and gout, and an increased prevalence of hyperuricemia, among this low-income, underrepresented population compared to the national average, as reported by the National Health and Nutrition Examination Survey (NHANES), conducted by the Centers for Disease Control and Prevention's (CDC) National Center for Health Statistics (NCHS) [10].

## Methods

This is a multisite cross-sectional study conducted at three different low-income senior residential communities spanning across the mid-Atlantic United States region. The study employed a community-engagement research approach. The number of total residents at these locations range from 167 to 201 individuals. The majority of residents at these locations fall into the age range of 62–79 years old. The average annual income of residents in these communities spans from $13,685 to $16,696. Inclusion criteria for participation included individuals of any gender, between the ages of 35 and 92, residing within the communities. This age range encompasses the totality of eligible participants at each location. The study was advertised to all community residents during an educational session. At these educational sessions, medical students who conducted the study described the goals of the study, the process of collecting health information, and the importance of awareness of chronic health conditions like diabetes and gout. All voluntary participants in this study were community residents and matched our inclusion criteria; no participants refused consent or were deemed ineligible. Exclusion criteria comprised individuals with a personal history of any of the following: cancer, organ transplantation, and current pregnancy.

Data from participants were collected during various meetings held from February to May of 2023. A total of 88/88 participants, representing diverse racial and ethnic backgrounds, were surveyed after obtaining informed consent. All participants signed a consent form approved by the Philadelphia College of Osteopathic of Medicine Institutional Review Board. First, participants responded to an electronic questionnaire covering dietary habits, a comprehensive personal and family medical history, social history, and current medications. Following the questionnaire, a medical student recorded participants’ anthropometric and vital measurements. Body fat percentage was measured using the Omron HBF-306C Handheld Body Fat Loss Monitor (OMRON HEALTHCARE, INC., Bannockburn, Illinois). Thirdly, medical students utilized commercially available lancets and strip-operated test kits to measure participants’ random non-fasting blood glucose, uric acid, and hemoglobin A1C. The glucose meter used was the Contour Next EZ Blood Glucose Monitoring System with Contour Next Blood Glucose Test Strips (Ascensia Diabetes Care, Parsippany, NJ). The uric acid meter used was the UASure II Uric Acid Meter Test Kit and Strips (ApexBio, Hsinchu City, Taiwan). The HbA1c testing device utilized was the A1CNow + HbA1c Professional Multi-Test HbA1c System (PTS Diagnostics, Inc., Whitestown, Indiana). Participants received educational materials, including informational packets. Subsequently, their blood glucose, uric acid, and HbA1c results were discussed. Individuals identified as at-risk were provided guidance on reducing their risk for diabetes and gout.

The collected data underwent analysis using a standardized linear regression model, with a 95% confidence interval. Further statistical analysis included chi square, cross-tabulation, and independent samples t-tests. Self-report was used to determine the diagnosis of T2D and gout. In addition, self-reported current use of anti-diabetic medication, as well as allopurinol, febuxostat, and colchicine, were considered to ascertain a diagnosis of diabetes and/or gout. A standardized linear regression was used to assess the association between T2D and gout (hyperuricemia), adjusting for traditional risk factors using adjusted odds ratios.

## Results

### Participant background: anthropometric data and pertinent medical history.

This study includes 62 female and 26 male participants with an average age of 68 ± 11 years. The average body weight was 193.9 ± 53.7 lbs, ranging from 106.8 to 380.0 lbs. The average height of participants was 64.8 ± 4.1 inches, resulting in an average Body Mass Index (BMI) of 32.2 ± 8.9, classified as Class 1 Obesity [11]. From our calculations, 17 (19%) participants measured a BMI within the normal range, whereas 27 (31%) were classified as overweight and 46 (52%) were classified as obese. The obese category was further sub-categorized into Class 1 (16), Class 2 (14), and Class 3 (16) obesity [9]. The average body fat percentage for all participants was 39.8% ± 9.6%, and the average waist circumference was 42 ± 8 inches (Table 1).

**Table 1:** Participant anthropometric data

Characteristic	Average Value ± SD
Male	26
Female	62
Age (years)	68 ± 11
Weight (lbs)	193.9 ± 53.7
Height (inches)	64.8 ± 4.11
Body Mass Index (BMI)	32.2 ± 8.9
Body Fat Percentage (%)	39.8 ± 9.6
Waist Circumference (inches)	42.0 ± 8.0

Among the participants, 97% reported having a current family physician. Regarding recent medical visits, 71 participants reported visiting their physician within the past 3 months, 7 within the past 6 months, 6 within the past year, 3 over a year ago, and 1 participant could not recall their last visit.

### Participant background: social and dietary habits

In addition to diabetes and gout, this population also experiences a variety of accompanying comorbidities. Of 88 participants, 87 reported having one or more chronic comorbid health condition(s). Present comorbidities include prediabetes, T2D, obesity, high blood pressure, high cholesterol, heart disease, and kidney disease. Specifically, 18 participants (20%) reported having prediabetes, 43 (49%) reported high blood pressure, 38 (43%) reported high cholesterol, 20 (23%) reported heart disease, and 10 (11%) reported kidney disease. Regarding diabetes, 29 participants who self-reported as diabetic are currently medicated, while 15 are not. Of the 87 participants with comorbidities, 17.2% (15 participants) reported having gout. Of these 15 participants, 26.7% (4 participants) reported being currently medicated for the condition.

Tobacco and alcohol are prevalent among this group of participants. In terms of smoking habits, 33 participants (38%) reported never smoking cigarettes, 33 (38%) reported smoking in the past but have since quit, 4 (5%) reported smoking about 4 packs of cigarettes monthly, 8 (9%) reported smoking about 4 packs weekly, and 8 (9%) reported smoking 1–2 packs daily. In the context of alcohol consumption, 23 (27%) participants reported never drinking, 25 (29%) reported drinking in the past but have since quit, 24 (28%) reported consuming about 4 drinks annually, 5 (6%) reported about 4 drinks monthly, 3 (3%) reported about 4 drinks weekly, 3 (3%) reported about 4 drinks daily, and 4 (5%) reported about 1–2 drinks daily. Two individuals chose not to report.

Dietary habits, including protein consumption, play a crucial role in blood serum urate levels. Among the participants, 72 (82%) reported consuming some form of protein daily, while 16 (18%) did not. In terms of frequency, 57 (65%) reported consuming protein once a day, 17 (19%) reported twice a day, 12 (14%) reported three times a day, and 2 (2%) reported never eating protein. When considering the sources of dietary protein, the average intake among the participants included 46 (52%) reporting eating chicken, 42 (48%) reporting seafood, 33 (38%) reporting red meat, and 12 (14%) reporting the use of protein supplements. In relation to gout symptoms specifically, 39 (44%) participants reported experiencing intense joint pain, 24 (27%) reported inflammation and redness of their joints, and 31 (35%) reported limited range of motion. Among those reporting joint pain, the most common locations included the knees (28 participants (32%)), back (12 participants (14%)), and hip (7 participants (8%)).

### Results of hemoglobin A1c and blood glucose measurements

Self-report was utilized to obtain diabetic status. From the collected samples of all participants, the average blood glucose level was 149.5 ± 71.7 mg/dL, and the average HbA1c reading was 6.2 ± 1.5%. These measurements together fell within the prediabetic range of 100 to 125 mg/dL for glucose levels and 5.7–6.4% for HbA1c [2]. The tests conducted confirmed diabetes in 34 participants and indicated diabetes in 12 individuals with no previous T2D diagnosis. HbA1c levels indicative of T2D (≥ 6.5%) were observed in 26 participants for both males and females. Additionally, 28.4% of participants had an HbA1c greater than 6.5, and 15.9% had a blood glucose level exceeding 200 mg/dL, which increases risk for the development of diabetes. Among the tested participants, 46.6% reported having a previous diagnosis of diabetes.

### Results of serum urate measurements

Self-report was utilized to obtain the history of gout. From the collected samples of all participants, the average uric acid level was 5.5 ± 2.2 mg/dL. Of these, 27 participants had hyperuricemia, or elevated urate levels greater than 7.0 mg/dL for males and greater than 6.0 mg/dL for females [3]. The tests indicated uncontrolled hyperuricemia and increased risk for gout flares in 5 individuals with a previous gout diagnosis, and indicated increased risk for gout and hyperuricemia-related comorbidities in 22 participants with no previous gout diagnosis. Among the tested participants, 17% of patients reported having a previous diagnosis of gout. At the time of testing, 30.7% of participants had elevated uric acid levels, a major risk factor for developing gout. The collected data is presented by gender in Table 2.

**Table 2:** Participants’ quantitative blood measurements

	Average Value ± SD	
Measure	Males	Females
Blood Glucose (mg/dL)	156.96 ± 75.88	146.35 ± 70.21
Hemoglobin A1c (%)	6.20 ± 1.33	6.23 ± 1.54
Serum Urate (mg/dL)	5.77 ± 2.27	5.32 ± 2.12

### Statistical analyses

**Fig. 1:**
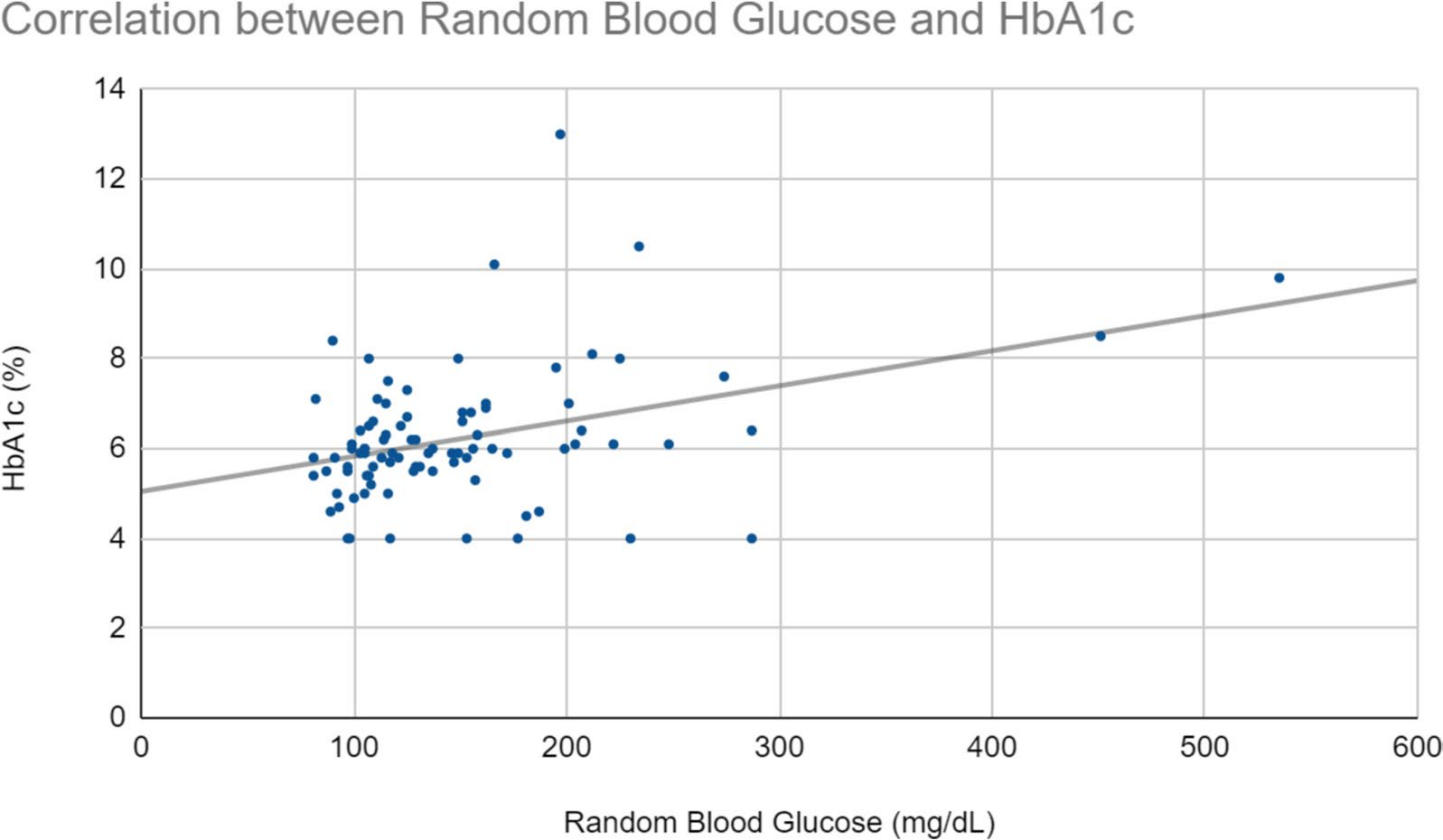
Correlation between Random Blood Glucose and Hemoglobin A1c levels in 88 participants. Analysis of HbA1c and random blood glucose data revealed a positive correlation between the two, with the regression equation Y = 0.009x + 5.045 and an R^2^ value of 0.218

**Fig. 2:**
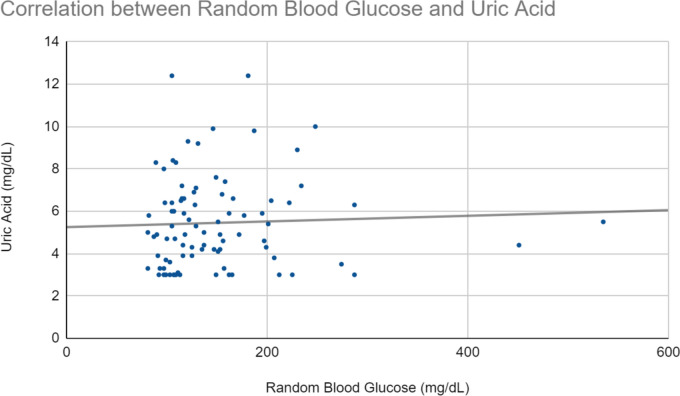
Correlation between Random Blood Glucose and Uric Acid levels in 88 participants. Analysis of uric acid and random blood glucose data revealed a weak positive trend between X and Y; however, this trend is not statistically significant. The regression equation Y = 0.003x + 5.373 and an R^2^ value of 0.008

A standardized linear regression was conducted to elucidate the predictive relationship between HbA1c and elevated uric acid levels. HbA1c levels were found to significantly predict uric acid levels, as diabetic HbA1c accounted for approximately 40% of the variance in uric acid, *R*^*2*^ = 0.401, *F*(1, 86) = 57.56, *p* < 0.001. The fitted regression model was: uric acid = 4.59 + 3.02*(HbA1c). Regression analysis of HbA1c and random blood glucose data revealed a positive statistically significant correlation between the two variables (Fig. 1). Regression analysis of uric acid and random blood glucose data revealed a weak positive trend, though not statistically significant (Fig. 2).

An independent sample t-test was performed to assess differences in uric acid levels between the high and low HbA1c groups. High HbA1c was defined as > 6.5%, low HbA1c was defined as < 6.5%, and hyperuricemia was defined as > 6 mg/dL in females and > 7 mg/dL in males. After adjusting for heterogeneity of variances across the two groups, the group of participants with high HbA1c had significantly higher levels of uric acid compared to the group of participants with low HbA1c, *t* (33.29) = − 6.47, *p* < 0.001. No significant differences were identified between high and low HbA1c groups relating to weight, BMI, and body fat.

A Pearson Chi-Square analysis was utilized to determine if a statistically significant association was present between identified variables. There were no significant associations between a T2D diagnosis and hyperuricemia, *χ*^*2*^ (1) = 0.003, *p* = 0.958, between red meat consumption and hyperuricemia, *χ*^*2*^ (1) = 1.47, *p* = 0.226, nor between an obese BMI (> 30.0) and hyperuricemia, *χ*^*2*^ (1) = 0.266, *p* = 0.606.

## Discussion

A diabetic HbA1c level predicted that a participant had hyperuricemia. 27 participants (30.7%) had levels of uric acid indicative of hyperuricemia, which is a risk factor of developing gout. This prevalence is higher than the national average, where only 20.1% of healthy adults experience asymptomatic hyperuricemia, and 3.9% of adults suffer from symptomatic gout flare ups [12], as reported by the NHANES [10]. In the elderly population, the prevalence of hyperuricemia is about 26.1% (ages 60–79) and 27.8% (ages 80 +) [10]. Additionally, as predicted, HbA1c was positively correlated with random non-fasting blood glucose. Our tests revealed diabetic blood glucose and HbA1c values in 34 participants and indicated increased risk of diabetes in 12 individuals with no previous T2D diagnosis. Additionally, our test indicated hyperuricemia in 5 individuals with a gout diagnosis, and indicated hyperuricemia in 22 participants with no previous gout diagnosis. It is important to acknowledge that the limited sample size of our study restricts our ability to draw definitive conclusions regarding significant differences between our cohort and the general population. Given that our sample may not be fully representative, the inclusion of even one or two additional non-hyperuricemic participants could potentially alter the observed differences. For comparative purposes, we reference NHANES data, which reports a national prevalence of hyperuricemia at 20.1% in the general population [10]. Our findings indicate a modest deviation from this national average, but due to the constraints of our sample size, further studies with larger, more representative cohorts are needed to draw more robust conclusions.

Study participants who smoked cigarettes were more likely to have hyperuricemia compared to nonsmokers. This correlation is most likely due to the behaviors associated with smoking and gout [13]. There is a strong lack of health education in this patient population, and many have been smoking since adolescence. In a seven-year longitudinal follow-up study of adult smokers, smoking predicted dietary behavior and alcohol use [13]. Heavy smokers were found to consume fewer vegetables, fruit, milk, and other dairy products. Additionally, alcohol consumption is one of the strongest facilitators of smoking. Alcohol has been shown to enhance the rewarding effects of nicotine by increasing neuronal firing rates and increasing dopamine [13]. 74% of the participants in this study reported consuming alcohol currently or in the past. 63% reported smoking cigarettes currently or in the past. This relationship may suggest why the patient population under study is consuming alcohol and smoking. Likewise, alcohol consumption is related to gout. Ethanol enhances adenine nucleotide degradation which over time leads to the inability of the kidneys to filter excess nitrogen content and leads to elevated serum uric acid [14]. The combination of alcohol, smoking, and dietary choices are all interrelated in the development of gout. In a similar vein, 24 participants reported smoking cigarettes currently or in the past with a diabetes diagnosis. This large overlap is most likely related to worsened glucose tolerance and insulin sensitivity in patients who are smokers. [15]. Likewise, nicotine has been shown to alter glucose homeostasis, indicating its contribution to the development of T2D.

One of the main objectives of this study was to investigate the relationship between diet and hyperuricemia. In this underserved population, a main proponent of poor diet came from a lack of education on the classification of food types such as protein, fat, and carbohydrates. Many individuals were unaware that the consumption of red meat, alcoholic beverages, and fatty foods could accelerate gout development due to high purine content and lactic acid accumulation [16]. In addition, a significant relationship was found to exist between obesity and elevated serum urate levels. Of the 46 participants who were obese, 13 had an elevated serum urate level (28%). Furthermore, of the 27 participants with elevated serum urate, 13 were obese (48%). These conditions may exist as the result of poor dietary and lifestyle habits. This finding emphasizes the need for proper education on lifestyle modifications to decrease the incidence of gout and its related comorbidities.

Gout is just one of many comorbidities that disproportionately affect senior adults throughout the world. Obesity, chronic kidney disease, hypertension, dyslipidemia, cardiac diseases, stroke, peripheral artery disease, and T2D often accompany gout in disease presentation [17]. The elevated serum urate can promote the occurrence and development of cardiovascular disease through multiple regulatory mechanisms, such as oxidative stress, inflammation, and insulin resistance. For instance, uric acid has been associated with atherosclerosis, as a significant elevation can cause overexpression of inflammatory markers such as nuclear factor κB (NF-κB) and vasoconstrictors that cause endothelial dysfunction [18]. The complex interplay of these coexisting conditions in senior individuals, especially those who lack access to health resources, emphasizes the importance of preventative community health screenings, outreach programs, and health education.

Although the association of gout or hyperuricemia with diabetes or with obesity has been a well-known phenomenon, this study showed a higher prevalence of gout and hyperuricemia among low-income elderly residents of Beacon Communities, compared to the national average [10]. This may stem from several factors. Limited access to healthy food, healthcare, and medications may contribute to poor management of conditions like obesity and diabetes, both of which are linked to elevated uric acid levels. Additionally, dietary patterns high in purines, sedentary lifestyles, and increased stress can exacerbate these conditions. The elderly in low-income areas may also face barriers to proper diagnosis and treatment, further increasing the risk of gout. These combined factors likely explain the higher prevalence in this community.

While our study highlighted the relationship between diabetes and gout in an underserved patient population, as well as the complex interplay of various socioeconomic factors in the development of these conditions, there were several limitations. A major limitation was that our tests were not confirmatory for gout and only indicated the possibility of hyperglycemia, which contributes to T2D. Another limitation was the use of non-fasting blood glucose and point-of-care HbA1c tests, which are not suitable for diagnostic screening for diabetes [19]. Additionally, the study's limitations include potential confounding factors that may not have been fully accounted for, such as variations in dietary habits, medication use, and access to healthcare.

Anti-diabetic medications have a blood glucose lowering effect through a variety of mechanisms including the alteration of intestinal glucose transport [20] and sodium-glucose cotransport inhibition. Additionally, insulin supplementation is essential for many individuals living with T2D, which influences blood glucose values [21]. These factors can influence the observed prevalence rates and should be carefully considered when interpreting the results. The use of various anti-diabetic and anti-hyperuricemia medications could have independently affected both blood glucose and serum urate levels, influencing the outcome measures. However, due to the unknown quantities and timing of medication administration for each participant, it is difficult to fully account for their effects on the results. We acknowledge this as a significant limitation of our study.

It’s important to consider the risk of information bias in this study, as inaccuracies in self-reported data can affect prevalence estimates. In future development, our sample size should be larger in order to draw more conclusive data. Similarly, as 70% of our participant population identify as female, it would be beneficial to investigate the prevalence of these conditions in additional male participants. Furthermore, we had 2 patients who were previously diagnosed with hemostatic disorders that could have skewed their hemoglobin readings. The cross sectional, single time point design can introduce intrasubject and intersubject variabilities. In a future study, data collection at various time points may glean a more comprehensive representation of this population. Additionally, controlling for participant anti-diabetic and anti-hyperuricemic medication use may more clearly elucidate the relationship between diabetes, gout, and low socioeconomic status without the influence of these medications. In expanding this research project, we aim to reach more diverse locations to compare urban and rural populations among underserved communities. Our objective was to compare the prevalence of hyperuricemia in individuals with T2D in the low socioeconomic status group to the national average. Given the limitations of our sample size, we are unable to make strong comparisons between low and not low socioeconomic status groups. Further research with larger and more representative samples would be necessary to explore these differences more thoroughly. Similarly, it would be beneficial to compare individual socioeconomic factors in developing these comorbidities.

## Conclusion

Senior diabetic individuals with low socioeconomic status face an increased risk of developing hyperuricemia and gout. It is imperative to implement preventive measures to address the current alarming trends. Our hypothesis is supported by the data collected, revealing that 30.7% of participants exhibited serum urate levels indicative of hyperuricemia, which is a risk factor for gout, surpassing the national prevalence of hyperuricemia (20.1%) [10].

Our findings further highlight smoking as a potential risk factor for hyperuricemia and emphasize the relationship between elevated uric acid levels and increased susceptibility to developing T2D. Notably, contrary to existing research, our sample did not reveal a correlation between red meat consumption and uric acid levels, underscoring the limitations posed by the small sample size. A significant 52% of participants in our study were identified as obese, potentially reflecting limited access to affordable and healthy food choices. Strategies to reduce uric acid levels include limiting purine-rich foods and those with high sugar content while incorporating regular exercise and patient education. Unfortunately, individuals at the highest risk often face barriers to accessing educational health resources and wholesome foods.

In light of these challenges, it becomes crucial to implement preventive educational initiatives focusing on diet and lifestyle. These efforts are essential to mitigate the incidence of gout and T2D in low-income elderly populations, addressing the disparities in health outcomes by enhancing access to knowledge and promoting healthier choices.

## Data Availability

The data supporting the findings of this study are available on request from the corresponding author. The datasets are stored in a institutional cloud storage service, and access can be granted following the appropriate data-sharing agreements.

## Abbreviations

HbA1c: Hemoglobin A1c
T2D: Type 2 diabetes
BMI: Body mass index
NHANES: National health and nutrition examination survey
